# The Impact of Phloem Feeding Insects on Leaf Ecophysiology Varies With Leaf Age

**DOI:** 10.3389/fpls.2021.625689

**Published:** 2021-07-16

**Authors:** Sylvain Pincebourde, Jérôme Ngao

**Affiliations:** ^1^Institut de Recherche sur la Biologie de l’Insecte, UMR 7261, CNRS, Université de Tours, Tours, France; ^2^Université Clermont Auvergne, INRAE, PIAF, Clermont-Ferrand, France

**Keywords:** *Aphis pomi*, herbivory, leaf age, leaf gas exchange, nitrogen content, photosynthesis, stomatal conductance

## Abstract

Herbivore insects have strong impacts on leaf gas exchange when feeding on the plant. Leaf age also drives leaf gas exchanges but the interaction of leaf age and phloem herbivory has been largely underexplored. We investigated the amplitude and direction of herbivore impact on leaf gas exchange across a wide range of leaf age in the apple tree–apple green aphid (*Aphis pomi*) system. We measured the gas exchange (assimilation and transpiration rates, stomatal conductance and internal CO_2_ concentration) of leaves infested versus non-infested by the aphid across leaf age. For very young leaves up to 15 days-old, the gas exchange rates of infested leaves were similar to those of non-infested leaves. After few days, photosynthesis, stomatal conductance and transpiration rate increased in infested leaves up to about the age of 30 days, and gradually decreased after that age. By contrast, gas exchanges in non-infested leaves gradually decreased across leaf age such that they were always lower than in infested leaves. Aphids were observed on relatively young leaves up to 25 days and despite the positive effect on leaf photosynthesis and leaf performance, their presence negatively affected the growth rate of apple seedlings. Indeed, aphids decreased leaf dry mass, leaf surface, and leaf carbon content except in old leaves. By contrast, aphids induced an increase in leaf nitrogen content and the deviation relative to non-infested leaves increased with leaf age. Overall, the impacts of aphids at multiple levels of plant performance depend on leaf age. While aphids cause an increase in some leaf traits (gas exchanges and nitrogen content), they also depress others (plant growth rate and carbon content). The balance between those effects, as modulated by leaf age, may be the key for herbivory mitigation in plants.

## Introduction

Plant gas exchanges are at the forefront of ecosystem functioning, as they are measurements of heat and mass exchange between the plant and the atmosphere. The impact of biotic and abiotic stressors on plant gas exchanges has received considerable attention, in particular for agricultural systems (Giron et al., 2018). The impacts on plant gas exchange of injuries caused by herbivore insects can be roughly of similar amplitude than the influence of climatic variables like temperature, irradiance, and humidity (Jarvis, 1976; Welter, 1989; Peterson, 2000). This comparison denotes the potential importance of herbivore injuries on the functioning of vegetation-atmosphere interactions. Nevertheless, any attempt to classify the impacts of herbivore insect species as negative or positive for leaf ecophysiology remains challenging because the direction of the impact depends on the nature of the system and insect feeding strategies (Welter, 1989).

The impacts of insect herbivory on photosynthesis are highly variable and depend on the exact insect–plant interaction. Most of the time, the loss of photosynthetic tissues following feeding by defoliating insects induces an increase in photosynthetic rate per unit area in the remaining leaf tissues, allowing the plant to compensate partially for herbivory (Welter, 1989). In other cases, herbivory induces a decrease in assimilation rate in the remaining leaf tissues (Zangerl et al., 2002). Large reductions in photosynthesis were also measured on leaves attacked by mesophyll feeders like spider mites (Welter, 1989; Haile and Higley, 2003) and stink bugs (Velikova et al., 2010). In phloem feeders like aphids, photosynthesis of the host plant can be dramatically lowered (Macedo et al., 2003), while sap feeders such as scale insects can induce an increase in leaf assimilation rate (Retuerto et al., 2004). Generally, an increase in photosynthesis following herbivory is interpreted as a strategy for the plant to compensate for the effect of the herbivore (Trumble et al., 1993). However, phloem feeders like aphids display the ability to strongly and actively reconfigure the leaf metabolism via effectors (Giron et al., 2018), which may annihilate the mitigation strategy of the plant. Effectors from aphids and spider mites, for instance, have been shown to suppress plant defense signaling and responses, thereby increasing the performance of the herbivores (Atamian et al., 2013; Naessens et al., 2015; Schimmel et al., 2017). The cascading consequences of this suppression for photosynthesis remain unclear.

The effects of insect herbivory on leaf stomatal conductance and transpiration rate are also quite variable. Insect injuries can cause an increase in water loss across the perimeter of the damaged tissues in soybean (Aldea et al., 2005). Both net photosynthesis and stomatal conductance in the remaining leaf tissues were not affected in this system involving defoliating beetle (*Popillia japonica*) and caterpillar (*Helicoverpa zea*). By contrast, Tang et al. (2006) indicated that both water stress, induced by the increased rate of water loss near the damaged tissues in Arabidopsis, and the reduced stomatal conductance in the tissues away from the injuries (by the Lepidoptera *Trichoplusia ni*) contributed to the inhibition of photosynthesis in the remaining leaf tissues. The general conclusion that can be drawn is that either assimilation and transpiration rates are affected concomitantly or photosynthesis is reduced while water loss increases. In the first case, the leaf efficiency (water use efficiency) remains at best constant if the plant compensates for the loss of tissues from herbivory. Full compensation is, however, rather rare (Peterson, 2000), but mitigation is possible and may contribute to plant tolerance against herbivores (Pincebourde et al., 2006).

The observed variability in the response of leaf gas exchange to insect herbivory remains difficult to explain. Herein, we argue that leaf age can be responsible for a significant part of this variability, following the suggestion from Trumble et al. (1993) that “the ages of the plants examined for compensation responses undoubtedly contribute to the observed variability in the literature.” Indeed, studies on the influence of leaf age on plant gas exchange offer promising insights. In general, photosynthesis and stomatal conductance decrease with leaf age mostly because the foliar nitrogen content is gradually reduced as the leaf is aging (Kositsup et al., 2010; Zhang et al., 2010) and also because mesophyll diffusion constraints photosynthesis more in older than in younger leaves (Niinemets et al., 2005). The influence of leaf age on photosynthesis does not depend on leaf longevity and instead relies on complex biochemical and structural dynamics (Mediavilla and Escudero, 2003; Pantin et al., 2012). Nutrients and defensive metabolites also vary with leaf age (Cao et al., 2018). Although most studies considered categories of leaf age (e.g., young versus old), more detailed works illustrated the rather subtle influence of leaf age on plant photosynthesis. Gas exchanges can gradually increase in very young leaves up to a maximum after which they decline (Ho et al., 1984; Guo and Lee, 2006; Zhang et al., 2008; Snider et al., 2009). Given the large effect of leaf age on plant gas exchange, it is therefore not surprising that leaf age interact strongly with other processes such as leaf response to heat stress (Snider et al., 2010; Marias et al., 2017), to increased atmospheric CO_2_ concentration (Katny et al., 2005) and to tropospheric ozone (Zhang et al., 2010). The influence of leaf age on the leaf gas exchange’s response to herbivore insects remains, however, largely underexplored. Here, we quantified the gas exchanges of leaves from plants attacked by an aphid across leaf age.

Our objective was to quantify the influence of leaf age on the amplitude of change in plant gas exchange following herbivore attack in the apple tree-green aphid (*Aphis pomi*) system. We surveyed apple seedlings during spring and summer in a greenhouse system such that the age of every leaf was known by the end of the growth period. A group of seedlings was infested by the apple green aphid to determine the leaf age preference of the aphid. We measured assimilation rate, stomatal conductance, internal CO_2_ concentration and transpiration for different leaf ages across this period. On leaves of about 30 days, the apple green aphid causes an increase in leaf transpiration and assimilation rates at a moderate infestation level (Pincebourde and Casas, 2019) but it also induces a decrease in these gas exchanges during early infestation stage (Cahon et al., 2018). We tested the hypothesis that gas exchange are enhanced in relatively young leaves but that infested leaves converge toward non-infested leaves as the leaf is aging. We further measured the impact of the aphid on leaf dry mass, leaf surface and leaf mass per area (LMA), nitrogen and carbon content, and plant growth rate to obtain a near-holistic assessment of the impact of the aphid on plant performance.

## Materials and Methods

### Study System, Design and Leaf Age

For all experiments, we used apple seedlings (*Malus domestica*, Golden cultivar) that were 1- to 3-year-old at the time of this work. These apple seedlings were issued from planting seeds obtained from a fruit tree seed bank (INRAE IRHS, Angers, France). Apple seedlings were planted in earthenware pots (11.5 cm in diameter) and watered every 2 days. A nutrient solution was added to the water once weekly (6% NO${}_{3}^{-}$, 6% P_2_O_5_, and 6% K_2_O, by volume). All seedlings grew in the same greenhouse in Tours, France (47°21′ N, 0°42′ E), until the time for experiments. Because all seedlings were pruned every year, they all had similar dimensions at the beginning of the experiment, before they initiate leafing (mean ± sd: height = 33.4 ± 4.5 cm; basal circumference of the trunk = 5.9 ± 2.6 cm). The green aphid *A. pomi* (Hemiptera: Aphididae) was collected in the apple orchard of La Morinière, close to our laboratory (47°09′ N, 0°35′ E; elevation: 95 m asl) in 2011. The aphids were subsequently reared on the apple seedlings in the greenhouse. We focused on the spring generation for all experiments. The experiments below occurred 2 years after the establishment of aphid populations on apple seedlings in the greenhouse, in 2013.

Early in 2013, before the seedlings started to produce leaves, the plants were split into two groups. The first group (“infested”; *N* = 18 seedlings) was left in the same greenhouse and was infested by the aphid (coming from the eggs apparent on the stems). The second group of plants (“non-infested”; *N* = 15 seedlings) was moved to another adjacent greenhouse to grow them without the presence of aphids. The stems were carefully cleaned with a soap solution to eliminate all the aphid eggs. These apple seedlings were inspected throughout the experiments to insure that they remain aphid-free. The two greenhouses were adjacent and exposed to the same environmental conditions. The climatic conditions inside the greenhouses varied daily but the range and global mean daily values were relatively stable during the experimental period in the “infested” and “non-infested” greenhouses, respectively: daily air temperature [range 14.5–38.5°C, global mean 24.4°C versus range 14.2–36.4°C, mean 24.3°C], daily relative humidity [range 29.5–95%, global mean 74% versus range 32.3–98.1%, mean 76.3%] and radiation load at the level of the plants was up to 875 W/m^2^ versus 853 W/m^2^. The air in the two greenhouses communicated via a large opening near the roof, ensuring that the atmosphere in the two units remained the same. The opening was covered with a fine-mesh net to impede the passage of any aphid. This design limited the potential for a greenhouse effect in our experiment.

Leaf age was determined by labeling the newly emerging leaves. Once a week, for all apple seedlings (infested or not by the aphid), the last emerging leaf was labeled with the date of emergence. At any point in time, the age of each leaf was retrieved with a precision of ±2 days by interpolating linearly between each weekly label (globally, seedlings produced between 2 and 5 leaves per week).

### Leaf Gas Exchanges

Assimilation rate (Amax), transpiration rate (Tr), stomatal conductance (gsmax), and internal CO_2_ concentration (Ci) were measured on both non-infested and leaves infested by the green aphid within the period June 2, 2013 to July 12, 2013. Leaf gas exchanges were measured with an infrared gas analyzer equipped with a 2 × 3 cm leaf chamber system (LI-6400, Li-Cor Inc., Lincoln, NE, United States) and an external light source (6400-02B, Li-Cor Inc.). Gas exchange was measured under optimal conditions for apple leaves (Pincebourde and Casas, 2006; Pincebourde et al., 2006; Massonnet et al., 2007): irradiance 1500 μmol/m^2^/s, leaf temperature 25°C, leaf water vapor deficit 1 kPa and CO_2_ concentration of 390 ppm in air. Leaves were allowed to equilibrate for 20–30 min before any measurements were taken, and data were discarded if stomatal conductance was not stable after 45 min. For infested leaves, the leaf surface was gently brushed with a fine pencil to eliminate the aphids from the surface, such that the gas exchange from the animals did not bias the measurements for the leaf. We sampled gas exchange across a large spectrum of leaf ages from 7 to about 100 days. During each measurement session (i.e., 3 days per week during the period), leaves of different age were selected to finish with a relatively balanced sampling (*N* = 25 leaves from 5 to 125 days, from *N* = 15 different seedlings; and *N* = 30 leaves from 5 to 82 days, from *N* = 16 different seedlings; for non-infested and infested plants, respectively). Across the period, between 1 and 2 (non-infested) and between 1 and 3 (infested) leaves from the same individual tree were used at different sessions and to catch different leaf ages.

### Plant Growth Rate and Aphid Densities

At the end of the experimental period (last week of July 2013), the growth rate of apple seedlings (*N* = 18 infested and *N* = 12 non-infested seedlings) was estimated from the relationship of leaf area accumulation across time. The size (maximal length and width) of each leaf was measured with a digital caliper to the nearest 0.5 mm. The leaf area of each leaf was calculated using an empirical relationship that was determined on a subset of leaves taken from all plants (linear regression for non-infested plants: leaf area = 0.6405 × length × width, all in cm, *N* = 121 leaves, *R*^2^ = 0.95, *P* < 0.001; for infested plants: leaf area = 0.6066 × length × width, all in cm, *N* = 166 leaves, *R*^2^ = 0.95, *P* < 0.001). In this subset of leaves (those leaves with a datum label), leaf area was determined by scanning them immediately after collection, and by measuring their surface area in ImageJ software v1.53e (Wayne Rasband, NIH, 1997)^1^. Using the labels for leaf emergence date, the accumulation of leaf area was regressed across time. The slope of the linear regression was used as an estimate of growth rate for infested and non-infested apple seedlings. The accumulation of leaf area across time was not strictly linear and therefore the slope estimate should be seen as a time-averaged growth rate (all the *R*^2^ for the linear regressions were >0.90 and >0.83 for non-infested and infested plants, respectively).

Finally, the number of aphids (nymphs and reproductive females) and the age/area of their host leaf were noted (using the same methods than above) at the end of the experimental period to analyze the preference of aphids relative to the age of their host leaf. We discriminated the larval stages (nymphs) from adults (females) based on the large difference in body size and the darker (green) coloration of adults.

### Leaf Nitrogen and Carbon Contents

At the end of the experimental period (last week of July 2013), leaves were collected to measure their nitrogen and carbon contents. For both infested (*N* = 18) and non-infested (*N* = 12) apple seedlings, we sampled all the leaves with a datum label (i.e., one leaf for each week of growth). The leaves were frozen in liquid nitrogen and freeze-dried, dry weighed and finally ground prior to biochemical analyses. The total N and C concentrations were determined using an EA 1112 Series elemental analyzer (Thermo Fisher). In total, 124 and 166 leaves were sampled on non-infested and infested plants, respectively. Then, the LMA was calculated from the ratio of leaf dry mass and leaf surface. We also calculated the nitrogen to carbon ratio (N/C).

### Statistical Analysis

The impact of aphids on plant growth was assessed by calculating the slope of the linear regression fitted on the total leaf area accumulated through time. The distribution of growth slopes of infested and non-infested plants was then compared running a simple ANOVA (with plant treatment as factor). The variables related to gas exchange (Amax, Tr, gsmax, and Ci), leaf dry mass, leaf surface, LMA, N and C contents, and N/C ratio were tested using an analysis of covariance (ANCOVA) with these response variables as dependent variable to compare between infested and non-infested plants (treatment as factor) with both leaf age and growth rate of individual plants as covariates. Normality and homogeneity of the variances were checked using the Lilliefors and the Levene’s test, respectively (transformation of the data was not necessary). We used the growth rate (slope of the linear regression explained above) of individual plants to account for the inter-plant variability for traits other than those that we measured. We took advantage of the ANCOVA to test the effect of treatment (infested versus non-infested) while accounting for the variability induced by both plant identity (growth rate) and leaf age; therefore the test on those covariates are seen as tests of the interaction with treatment. We also checked for the normality of the residuals from the ANCOVA model fit using a Lilliefors test after the ANCOVA run. A LOWESS smoother was applied to obtain an estimate of the trends. The water use efficiency was assessed using simple linear regressions on the relationship between assimilation rate and transpiration rate. The slopes of these regressions were used as an indicator of a change in the water use efficiency of the leaf under green aphid attack. All statistics were computed using SYSTAT 13.1 (Systat Software Inc., San Jose, CA, United States).

## Results

**FIGURE 1:**
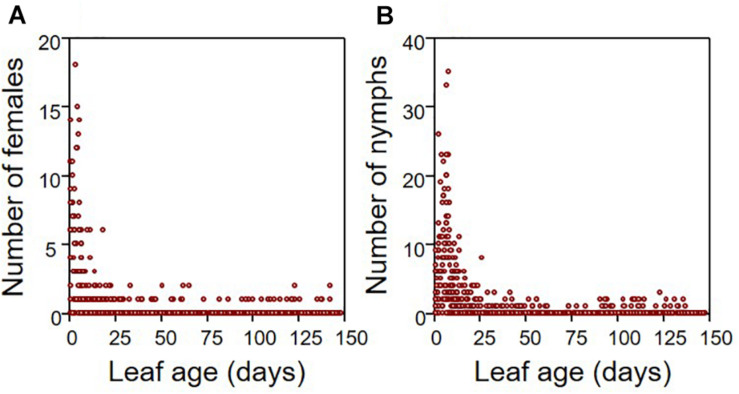
Distribution of aphids according to leaf age for both adult females **(A)** and nymphs **(B)**. These distributions were drawn from the number of aphids counted on each leaf of the 18 infested apple seedlings that were surveyed (total number of leaves = 847 leaves).

**FIGURE 2:**
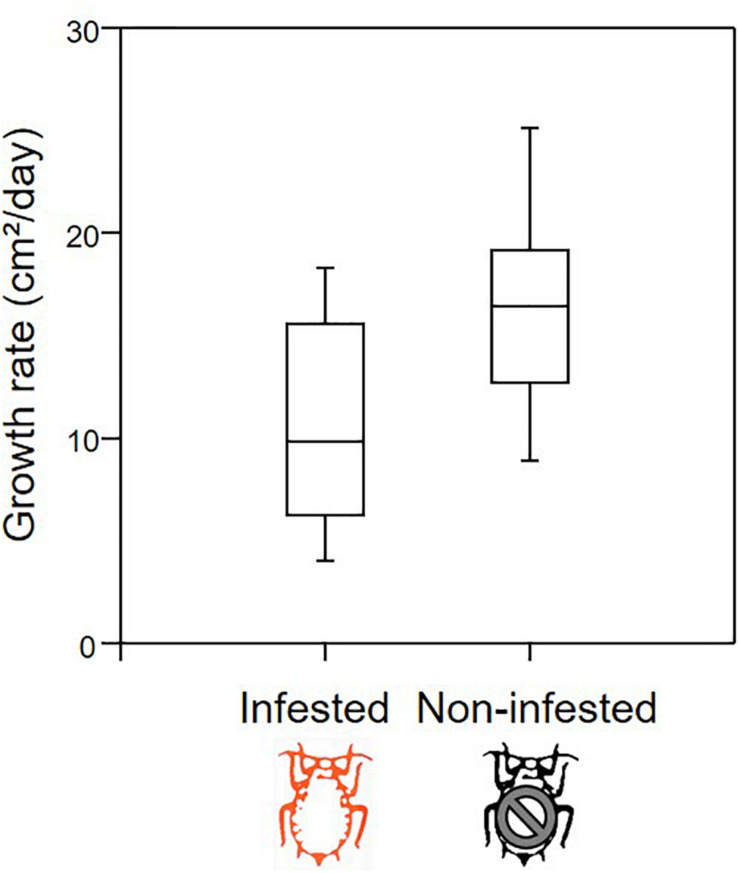
Box plot representation of growth rate (leaf area in cm^2^ per day) for infested (*N* = 18) and non-infested (*N* = 12) apple seedlings.

The exhaustive sampling at the end of the experiment indicated that aphids preferentially locate themselves on young leaves, both for females and juveniles (from *N* = 18 infested apple seedlings, *N* = 847 leaves). The vast majority of individuals were observed on leaves of <30 days (Figure 1). Throughout the growing period, we observed that aphids migrated regularly toward the recently emerged leaves at the tip of the stem, which displayed intermediate values for most leaf traits (Supplementary Figure 1). Therefore, the distributions measured at the end of the period may illustrate the location of aphids throughout the growing season. The growth rate of infested seedlings (*N* = 18 individuals) was on average 35% lower than that of non-infested (*N* = 12 individuals) seedlings (Figure 2; ANOVA: *F*_1,28_ = 10.655, *P* = 0.003).

**FIGURE 3:**
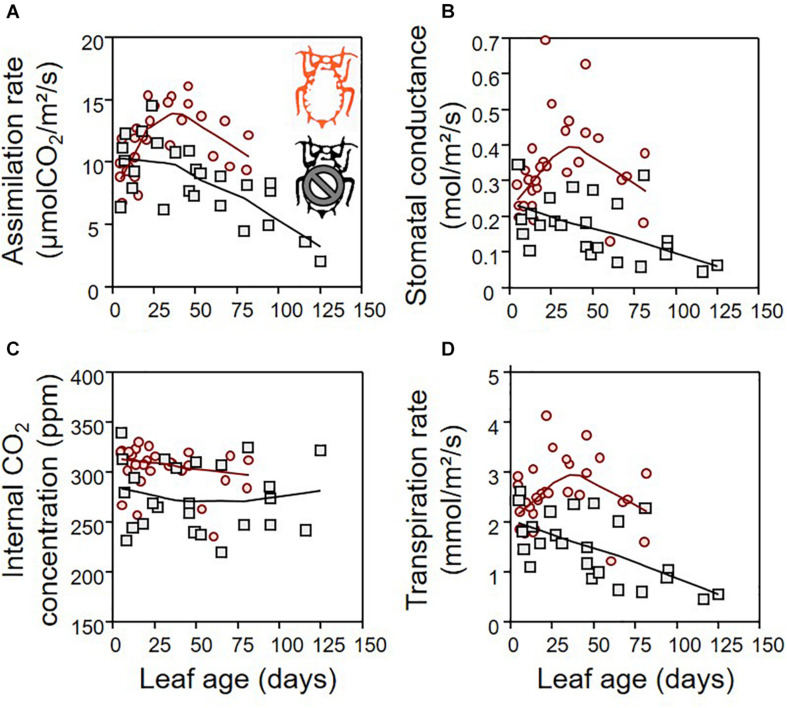
The effect of aphids on plant gas exchange depending on leaf age: leaf assimilation rate **(A)**, stomatal conductance **(B)**, internal CO_2_ concentration **(C)**, and transpiration rate **(D)**, for infested (red) and non-infested (black) plants. For the sake of visualization, a LOWESS smoother (0.8 tension) was applied on each data cloud.

**FIGURE 4:**
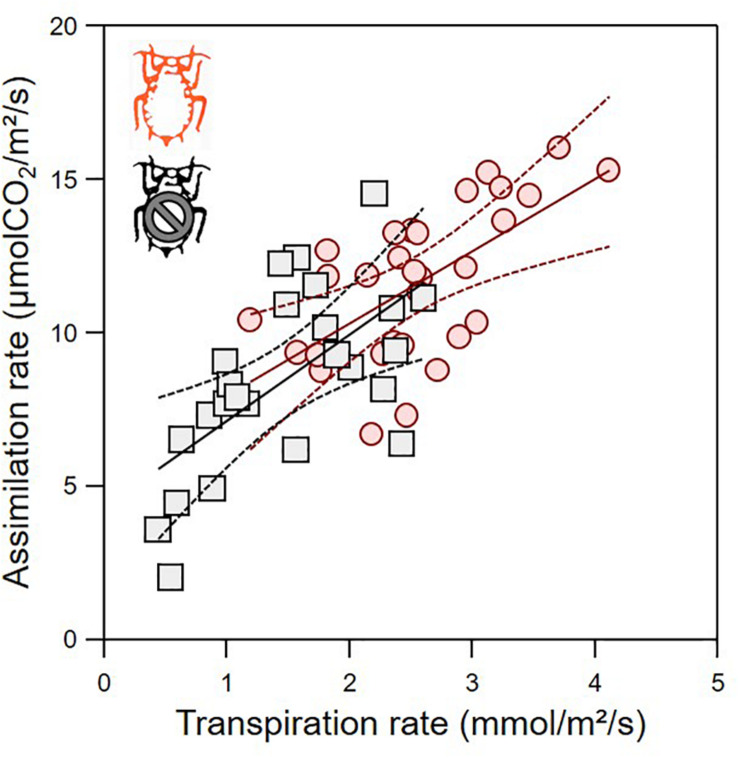
Optimality assessment of gas exchange for infested (red) and non-infested (black) leaves from the plot of leaf assimilation rate as function of transpiration rate. Lines are the linear regressions (full line) with their 95% confidence interval (dashed lines).

The green aphid largely influenced leaf gas exchanges in a way that depends on leaf age (Figure 3). The leaf assimilation rate (Amax), transpiration rate (Tr), stomatal conductance (gsmax), and internal CO_2_ concentration (Ci) differed between infested and non-infested plants when controlling for leaf age variability (Table 1; ANCOVA: *P* < 0.015 for all). Leaf age influenced Amax and Tr (Table 1; ANCOVA: *P* < 0.019 for both), but not gsmax and Ci (Table 1; ANCOVA: *P* > 0.05 for both). The growth rate of apple seedling impacted the measure of Amax and gsmax (Table 1; ANCOVA: *P* < 0.014 for both), but not that of Tr and Ci (Table 1; ANCOVA: *P* > 0.05 for both). Globally, the green aphid caused an increase in assimilation rate, transpiration rate and stomatal conductance up to leaf age of about 25 days after which these physiological variables decreased at a similar rate than in non-infested plants. The internal CO_2_ concentration was slightly but significantly higher in attacked leaves compared to intact plants. Finally, the leaf assimilation rate increased linearly with its transpiration rate for both infested (linear regression: *F*_1,28_ = 17.329, *P* = 0.001) and non-infested plants (linear regression: *F*_1,23_ = 14.986, *P* = 0.001) (Figure 4). The slope of increase was slightly lower for infested compared to non-infested plants (2.348 versus 2.794, respectively), and as a result the mean water use efficiency (ratio of assimilation and transpiration rate) was slightly lower in infested leaves than in non-infested leaves (ANOVA: *F*_1,53_ = 12.67, *P* = 0.001).

**TABLE 1:** Statistics of the analysis of covariance (ANCOVA) with the dependent variables (Amax, maximal assimilation rate; Gsmax, maximal stomatal conductance; Tr, leaf transpiration rate; Ci, internal CO_2_ concentration; Dry mass, the dry mass of the leaves; Leaf surface, the leaf area of the leaves; LMA, the leaf mass per area; Nitrogen, the leaf nitrogen content; Carbon, the leaf carbon content; N/C ratio, the ratio of nitrogen to carbon content) and the source of the effects, with treatment as factor (infested versus non-infested plants) and leaf age and plant growth rate as covariates.

Variable	Source	Type III SS	df	*F*-ratio	*P*-value
**Amax**	Treatment	123.442	1	20.226	**<0.001**
	Leaf age	36.771	1	6.025	**0.018**
	Growth rate	40.791	1	6.683	**0.013**
	Constant	287.941	1	47.178	**<0.001**
	Error	311.266	51		
**Gsmax**	Treatment	0.326	1	29.056	**<0.001**
	Leaf age	0.028	1	2.526	0.118
	Growth rate	0.05	1	4.442	**0.04**
	Constant	0.146	1	13.027	**<0.001**
	Error	0.572	51		
**Tr**	Treatment	11.777	1	31.239	**<0.001**
	Leaf age	2.561	1	6.794	**0.012**
	Growth rate	1.178	1	3.124	0.083
	Constant	14.011	1	37.165	**<0.001**
	Error	19.227	51		
**Ci**	Treatment	5397.436	1	6.668	**0.013**
	Leaf age	649.437	1	0.802	0.375
	Growth rate	16.335	1	0.02	0.888
	Constant	360056.358	1	444.844	**<0.001**
	Error	41279.346	51		
**Dry mass**	Treatment	0.018	1	4.733	**0.03**
	Leaf age	0.21	1	56.457	**<0.001**
	Growth rate	0.159	1	42.856	**<0.001**
	Constant	0.187	1	50.156	**<0.001**
	Error	1.064	286		
**Leaf surface**	Treatment	1.213	1	0.009	0.923
	Leaf age	10131.622	1	77.989	**<0.001**
	Growth rate	6466.103	1	49.774	**<0.001**
	Constant	3442.911	1	26.502	**<0.001**
	Error	36764.618	283		
**LMA**	Treatment	1220.537	1	8.733	**0.003**
	Leaf age	268.316	1	1.92	0.167
	Growth rate	197.898	1	1.416	0.235
	Constant	81006.442	1	579.622	**<0.001**
	Error	39551.325	283		
**Nitrogen**	Treatment	0.982	1	13.176	**<0.001**
	Leaf age	2.362	1	31.707	**<0.001**
	Growth rate	0.004	1	0.049	0.825
	Constant	78.851	1	1058.459	**<0.001**
	Error	21.306	286		
**Carbon**	Treatment	35.46	1	19.443	**<0.001**
	Leaf age	115.383	1	63.266	**<0.001**
	Growth rate	3.599	1	1.974	0.161
	Constant	44739.755	1	24531.276	**<0.001**
	Error	521.602	286		
**N/C ratio**	Treatment	0.001	1	19.863	**<0.001**
	Leaf age	0.002	1	52.551	**<0.001**
	Growth rate	0	1	0.118	0.731
	Constant	0.038	1	1106.818	**<0.001**
	Error	0.01	286		

*For each variable, the statistics display the Type III sum of square (Type III SS), the degrees of freedom (df), the Fisher test (*F*-ratio), and the corresponding *P*-value (in bold when significant with a threshold at 0.05).*

**FIGURE 5:**
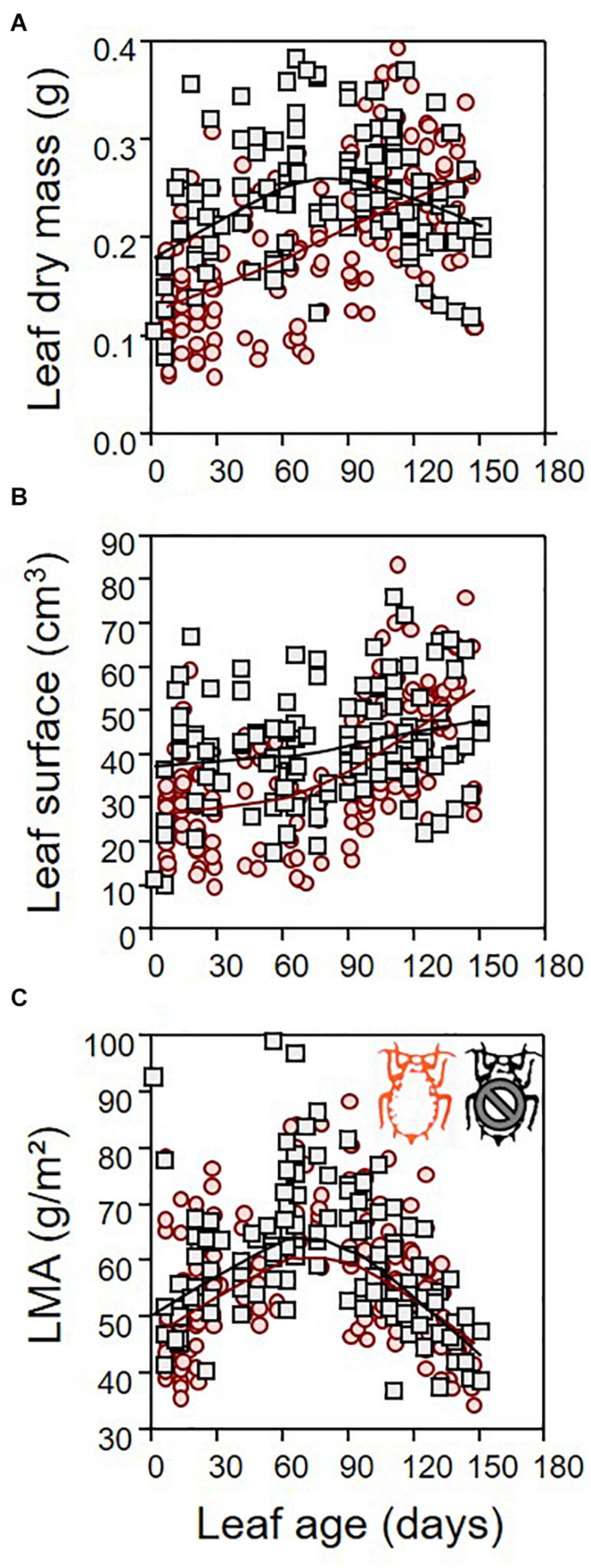
Leaf allometry. Leaf dry mass **(A)**, leaf surface **(B)**, and leaf mass per area (LMA) **(C)** as function of leaf age for both infested (red) and non-infested (black) plants. For the sake of visualization, a LOWESS smoother (0.8 tension) was applied on each data cloud.

**FIGURE 6:**
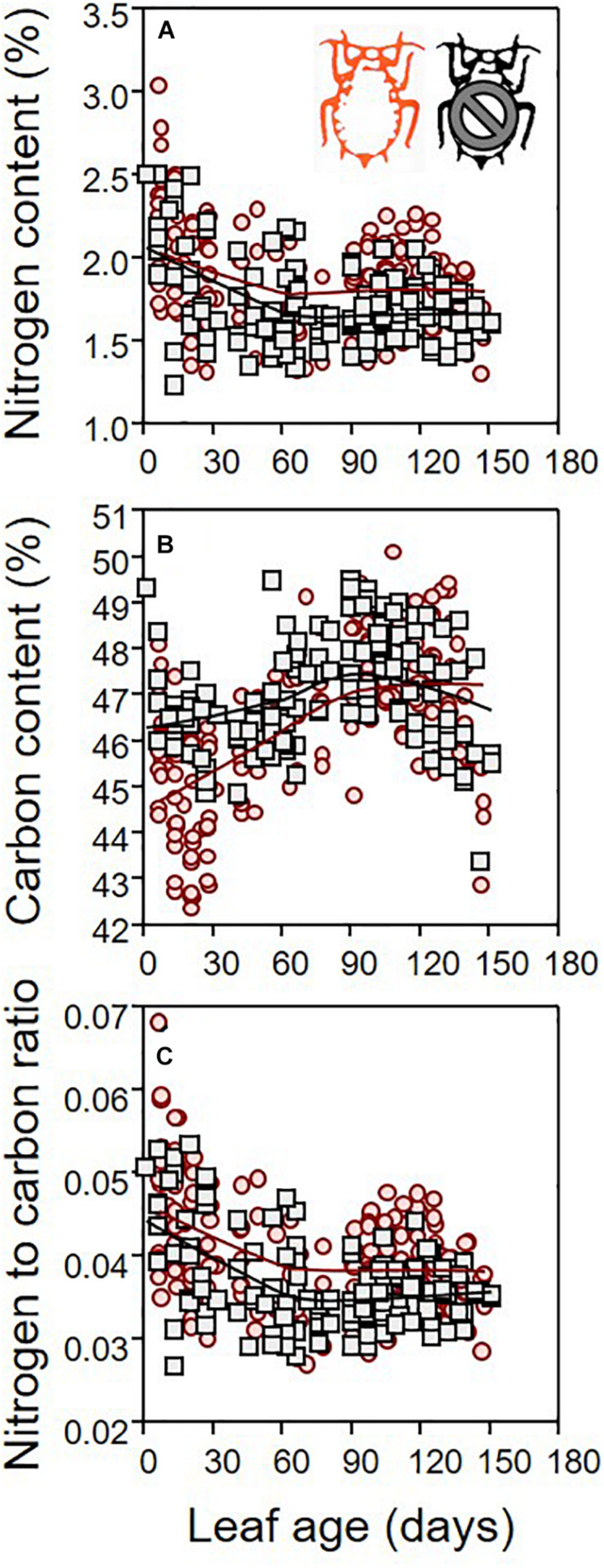
Nitrogen **(A)** and carbon **(B)** contents (% of leaf dry mass), and nitrogen to carbon ratio **(C)** of leaves sampled on infested (red) and non-infested (black) plants, as function of leaf age. For the sake of visualization, a LOWESS smoother (0.8 tension) was applied on each data cloud.

Leaf dry mass differed between infested and non-infested leaves (Table 1; ANCOVA: *P* = 0.03) depending on both leaf age (Table 1; ANCOVA: *P* < 0.001) and seedling growth rate (Table 1; ANCOVA: *P* < 0.001). Leaf dry mass was higher in non-infested leaves except in relatively old leaves, which became lighter than infested leaves (Figure 5A). By contrast, leaf surface was similar in infested compared to non-infested leaves (Table 1; ANCOVA: *P* > 0.05) but varied with leaf age and growth rate (Table 1; ANCOVA: *P* < 0.001) (Figure 5B). As a result, the LMA of infested plants was only slightly lower than that of non-infested plants (Table 1; ANCOVA: *P* = 0.003) and it was not influenced by leaf age and growth rate (Table 1; ANCOVA: *P* > 0.05) (Figure 5C). Nitrogen and carbon contents differed between infested and non-infested leaves (Table 1; ANCOVA: *P* < 0.001) and varied across leaf age (Table 1; ANCOVA: *P* < 0.001), but seedling growth rate did not impacted them (Table 1; ANCOVA: *P* > 0.05). Globally, infested leaves contained more nitrogen and less carbon than non-infested leaves (Figure 6). However, the carbon content of infested leaves converged toward that of non-infested-leaves as the leaves were aging. By contrast, the nitrogen content of infested and non-infested leaves were similar for young leaves, and the deviation increased with leaf age. As a result, the nitrogen to carbon ratio was slightly higher in infested leaves compared to non-infested plants, and the difference was modulated by leaf age (Table 1; ANCOVA: *P* < 0.001 for both) (Figure 6C).

## Discussion

Herbivore insects generate multiple biochemical, physiological, and ecological responses in the plant they attack. In particular, the impacts of herbivores on leaf gas exchange have been documented for a variety of insect taxa (Welter, 1989; Pincebourde and Casas, 2019). Nevertheless, the potential role of leaf age in modulating these effects has never been detailed. Our results indicate a strong interaction between leaf age and herbivory. The apple green aphid enhances assimilation rate, stomatal conductance and transpiration rate in apple leaves until about the 30th day of the leaf after which the aphids have left their leaf to migrate upward to younger leaves. The gas exchange then decreased gradually in leaves of age >30 days but they never meet the low levels of non-infested old leaves. This interaction was modulated by the growth rate of the apple seedlings, illustrating the importance of inter-individual variability in tree performance. The positive effect of the aphid on photosynthesis should balance somehow the negative incidence of aphid infestation on the plant growth rate and dry matter of leaves. When feeding on the phloem, aphids inevitably collect nutrients and carbon that are not available to contribute to plant growth. Globally however, the dry matter content and allometry of old infested leaves tend to converge toward the phenotype of non-infested plants, as the aphids do not feed on them anymore, but it takes almost 90 days for those leaves to converge. We found that aphids are preferentially located on young leaves (less than 25 days) at the end of our experimental period, but a more detailed and continuous monitoring of aphids is necessary to establish clearly the correlation between aphid presence and leaf gas exchange responses.

The apple green aphid induces an increase in assimilation rate when it attacks young leaves. It takes several days before the photosynthetic activity of infested leaves clearly starts to deviate from non-infested leaves. Then, the maximal deviation (about +50%) was observed at a leaf age of about 30–40 days. This result contrasts with the view that sap and phloem feeders have an almost universal negative impact on photosynthesis in woody plants (Zvereva et al., 2010), but adds up to previous studies indicating similar increase in assimilation rate after feeding from insects (Collins et al., 2001; Retuerto et al., 2004; Frier et al., 2012). Nevertheless, enhanced photosynthesis after herbivore feeding is not the rule among aphids (Meyer and Whitlow, 1992; Macedo et al., 2003). It seems that cell sap feeders generally reduce assimilation rate while the influence of phloem feeders (like the apple green aphid) is often null or positive (Zvereva et al., 2010; Frier et al., 2012). It remains unclear if the diversity of those effects relates to the diversity of effector/elicitor interactions used by insects and plants, respectively (Giron et al., 2018), especially given that the dynamics across leaf age of plant metabolites under insect attack remain to be characterized for most systems. However, the direction and amplitude of these effects on photosynthesis depends also on environmental and biotic parameters. Our experiments occurred under greenhouse climatic conditions that are somewhat more variable and hotter/wetter than in outdoor environments, and more research is needed to verify the strength of leaf age in modulating the plant-aphid interaction in more natural contexts. The infestation pattern certainly matters as well for the response of assimilation rate. For example, a previous study showed that the leaf temperature increases within the first 3 days after the apple green aphid started to feed on the apple leaf (Cahon et al., 2018), suggesting lower transpiration rate and stomatal conductance, and probably lower assimilation rate during early infestation on middle-aged leaves. It is unknown whether aphid females start infesting plants by attacking immediately the youngest leaves. Finally, we cannot exclude a covariation between leaf age, leaf allometry (surface, dry mass, and LMA) and aphid density across the season. Our design cannot detect such effect since aphids were not monitored throughout the experimental period. However, a higher growth rate of apple seedlings tended to increase even further the assimilation rate, suggesting that the effect of the phloem herbivore on photosynthesis was amplified by the capacity of the individual seedling to perform better. We hypothesize that the plant and the aphid drive each other at some point, thereby increasing both plant performance and aphid population growth. More work is needed to clarify this point.

Compensatory mechanisms are often suggested to explain the few cases of an increase in leaf assimilation rate following herbivore attack. At least two mechanisms were proposed to explain such increase in photosynthesis (Trumble et al., 1993). First, the feeding activity of the herbivore may decrease the resistance to CO_2_ diffusion across the mesophyll tissues and/or decrease the amount of starch accumulated within the leaf tissues–both factors normally inhibits photosynthesis. Indeed, the slightly higher intercellular CO_2_ concentration within the apple leaves infested by the apple green aphid (while assimilation is increased at the same time) supports this idea that the internal resistance to diffusion is lowered (Reddall et al., 2004). Second, the aphid itself may function as a new sink thereby increasing the photosynthetic activity of the attacked leaf. The increase in nitrogen content in the leaves attacked by the apple green aphid supports this sink hypothesis as shown in other systems (Syvertsen et al., 2003; Urban et al., 2004). The two mechanisms are not mutually exclusive and can also combine with other compensatory strategies at the cellular and biochemical levels (Trumble et al., 1993). Furthermore, in the case of interactions involving phloem feeders that reconfigure the leaf metabolism, the mitigation strategy could be annihilated by the effectors used by the herbivore. It remains challenging however to disentangle the effects of plant induced response to insect damage and the effects from insect manipulation of the leaf metabolism (Giron et al., 2018).

The apple green aphid also led to an increase in leaf transpiration rate, concomitantly to the increase in photosynthesis. The relationship between assimilation rate and transpiration rate (Figure 4) can be used as a proxy of the efficiency of the leaf to assimilate the highest amount of carbon (CO_2_) while limiting the water loss (instantaneous water use efficiency). Our result indicates that the leaf attacked by the green aphid is on the same “optimal” trajectory than non-infested leaves. In other herbivores (e.g., leaf miners), this relationship can be modified to the point that the infested leaf becomes even more efficient (Pincebourde et al., 2006). The aphid still have overall negative impact on the plant performance because its growth rate is lower, but our result suggest that these negative impacts can be mitigated by other effects that allow the plant to perform at a near-optimal level. The best examples of herbivory mitigation are in the leaf miner feeding guild (Raimondo et al., 2003; Pincebourde et al., 2006), which involves complex interactions with cytokinin production or accumulation at the mining location and a bacteria as a third partner (Giron et al., 2007, 2018). These effects of herbivory on leaf gas exchange could feedback on the ecophysiology of the insect pest itself (Van Loon et al., 2005). In particular, the leaf temperature variations following a change in leaf transpiration can influence the insect feeding and developmental performances (Pincebourde and Woods, 2012; Caillon et al., 2014; Pincebourde and Casas, 2015; Pincebourde et al., 2017; Ma et al., 2021). Aphids also host a diverse community of endosymbionts (Oliver et al., 2010), some of which can induce changes in plant volatile emission (Frago et al., 2017), but currently their indirect influence on the plant gas exchange are not known.

The influence of leaf age on the apple leaf–apple green aphid relationship is remarkable. Our results indicate that the aphid directs young leaves on a different ecophysiological path with a slightly lower instantaneous water use efficiency. Previous studies indicated a dynamics of leaf gas exchange (in intact plants) across leaf age (Ho et al., 1984; Guo and Lee, 2006) but these studies treated categories of leaf age (or leaf age classes) instead of analyzing it as a continuous variable. Our continuous analysis unravels the subtleties of the interaction between leaf age and leaf gas exchange, and more importantly how herbivory modify these links. When looking at very young leaves, the effect of herbivory can hardly be detected from gas exchange measurement alone. By contrast, the impact of herbivory on carbon content is already important early in the life of a leaf, certainly because the aphids are consuming most of the starch that contains the non-structural carbon. In old leaves, when aphids have already migrated upward, the carbon content (and dry mass) comes back to the level of non-infested leaves but gas exchange patterns still differ and the nitrogen to carbon ratio never gets back to the level of non-infested plants. Indeed, the leaf seems to compensate for the presence of an additional sink (the aphid) by increasing its surface relatively more than its dry mass, but our results indicate that this compensation may occur only in relatively old leaves (>70 days) when the aphids have already left to move upward to feed on younger leaves. This is coherent with the concept that the susceptibility of plant to stressors (including herbivory) is highest at the transition, when the leaf is aging, from metabolite sinks to metabolite sources (Coleman, 1986), but in our study system the herbivore may extend the metabolite sink stage of the leaf. Both aphid residence time and density are likely to modulate these dynamics across leaf age. By comparing the age distribution of the leaves used as hosts by the aphids at the end of the experimental period and the age range corresponding to the increasing trajectory of assimilation rate, we propose that photosynthesis is promoted as long as the aphid remains on its leaf. Therefore, we hypothesize that the leaf assimilation rate may reach even higher values if one constrains the aphid population to remain on the same leaf for a longer period. Experimental evidence are lacking to support this hypothesis.

The effects of herbivore insects on plant gas exchange can be subtle and vary with leaf age. Leaf age adds some complexity to an already quite sophisticated interaction since aphids largely influence the chemistry, physiology and ecology of their host plants (Giron et al., 2018). It remains challenging however to disentangle between the influence of the plant defenses (e.g., leaf secondary compounds; Nishida, 2014) and the herbivore effectors (Smith and Boyko, 2007), and we can suspect that both covary across leaf age. Our study system involves a temperate deciduous tree and as such, the leaf longevity remains relatively short (less than 9 months). Leaf lifespan is an important driver of insect-plant interaction dynamics (Zhang et al., 2017). Currently, no study has attempted to follow the response of plant gas exchange as function of leaf age in tropical plants, which display much higher leaf longevity than temperate woody species (Xu et al., 2017). Herbivory mitigation at the level of plant gas exchange may provide explanation to how plants could support moderate pressure from herbivores in systems with long leaf life span. More generally, our study suggests that leaf age modulates the interaction between plants and insects at the ecophysiological level. The control or standardization of leaf age is therefore required in any ecophysiological study investigating the impact of insect herbivores on leaf traits. The main challenge for future studies remain to integrate the impact of herbivores on leaf gas exchanges/traits, the relative importance of both constitutive and inducible plant defenses, the variability in the effectors used by herbivores, and the modulation of these interactions by leaf age.

## Data Availability Statement

The original contributions presented in the study are included in the article/Supplementary Material, further inquiries can be directed to the corresponding author/s.

## Author Contributions

SP designed the research. Both authors performed the research, analyzed the data, wrote the manuscript, and agreed on the submitted version.

## Conflict of Interest

The authors declare that the research was conducted in the absence of any commercial or financial relationships that could be construed as a potential conflict of interest.

## Footnotes

1 https://imagej.nih.gov/ij/

## Supplementary Material

We provide the raw data used in our study as Supplementary Material. Two datasets are provided. The file “Dataset_gas exchange” reports maximal photosynthesis (Amax, μmol/m^2^/s), maximal stomatal conductance (gsmax, mol/m^2^/s), internal CO_2_ concentration (Ci, ppm), and leaf transpiration rate (Tr, mmol/m^2^/s) as function of leaf age (days since emergence) and for each category of plant (non-infested versus infested by the apple green aphid). The dataset “Dataset_mass_N_C” provides leaf dry mass (g), nitrogen content (% of leaf dry mass), carbon content (% of leaf dry mass), the nitrogen to carbon ratio (N/C), leaf mass per area (LMA, g/m^2^) and leaf surface (cm^2^) as function of leaf age (days since emergence) and for each category of plant (non-infested versus infested by the apple green aphid).
