# Supplementary material for: The Impact of Phloem Feeding Insects on Leaf Ecophysiology Varies With Leaf Age
Source: Front Plant Sci. 2021 Jul 16;12:625689. doi: 10.3389/fpls.2021.625689 (PMC8322987; doi:10.3389/fpls.2021.625689)
Supplement: Supplementary file 1 [file Data_Sheet_1.zip › Supplementary Figure 1.docx]

**Supplemental material – Figure S1**

The impact of phloem feeding insects on leaf ecophysiology varies with leaf age

By Sylvain Pincebourde & Jérôme Ngao

**Fig S1.** The relationship between the number of aphids counted on leaves at the end of the experimental period and several leaf traits: the leaf area, leaf dry mass, nitrogen content and carbon content. No clear relationship emerged between these variables. The largest number of aphids where found on leaves displaying intermediate values for all traits.
